# Microscopic Malaria Infection and Its Determinants in Urban and Rural Populations Living in South-East Gabon

**DOI:** 10.1155/japr/8263358

**Published:** 2024-12-26

**Authors:** Jean-Claude Biteghe-Bi-Essone, Roméo Karl Imboumy-Limoukou, Steede-Seinnat Ontoua, Nick Atiga, Nancy Mbani-Mpega, Lady Charlène Kouna, Jean Bernard Lekana-Douki, Lydie Sandrine Oyegue-Liabagui

**Affiliations:** ^1^Unit of Evolution, Epidemiology and Parasitic Resistances, Franceville International Medical Research Centre, Franceville, Gabon; ^2^Research Laboratory, Regional Doctoral School in Tropical Infectiology, Franceville, Gabon; ^3^Department of Parasitology-Mycology, Libreville University of Health Sciences, Libreville, Gabon; ^4^Laboratory of Molecular and Cellular Biology, Masuku University of Science and Technology, Franceville, Gabon

**Keywords:** asymptomatic, attitude and practice, *Plasmodium*, rural, urban

## Abstract

A better understanding of malaria epidemiology in both asymptomatic and symptomatic individuals is essential for developing strategies to control the disease. This study was conducted to determine *Plasmodium* infection prevalence and its associated factors among people living in Franceville (urban area) and in the villages of Pana and Mvengue (rural areas) in south-east Gabon between April and July 2022. This cross-sectional study was conducted among all consenting residents of Franceville, Mvengue, and Pana between April and July 2022. After obtaining informed consent, *Plasmodium* sp. infection was screened by microscopy, and a structured questionnaire was developed to record sociodemographic data, attitudes, and practices regarding malaria. A total of 976 participants were included, with 491 in urban areas and 485 in rural areas. The overall prevalence of *Plasmodium* sp. infection was 21.62% (211/976; 95% confidence interval (CI) [19.15–24.31]). The prevalence was highest in children aged 6–11 years. In urban areas, the prevalence was 19.35% (95/491; 95% CI [16.10–23.07]), and 96.84% of infections were asymptomatic. The most infected age group was 18–23 years. In rural areas, the prevalence was 23.92% (116/485, 95% CI [20.34–27.91], and 93.97% (109/116) of infections were asymptomatic. Socioeconomic characteristics, attitudes, and practices towards *Plasmodium* sp. infection were not associated with a risk of asymptomatic malaria infection. This study highlights the importance of asymptomatic *Plasmodium* sp. infection in south-east Gabon and the need for control strategies adapted to different areas and age groups. Detection and treatment of asymptomatic carriers could be an important lever for malaria control and elimination in the country.

## 1. Introduction

Malaria is a parasitic disease transmitted by infected bites of *Anopheles* mosquitoes. Malaria is particularly formidable in tropical areas where *Plasmodium falciparum* exists and remains the deadliest parasitic disease for human beings. According to the latest report from the World Health Organization (WHO), the number of malaria-related deaths in 2022 amounted to 426,000, representing a decrease in the number of deaths compared to the previous year [1]. *Plasmodium* infection could be asymptomatic or cause febrile symptoms, often mild but sometimes fatal, especially in children under 5 years of age and pregnant women [1, 2]. Asymptomatic malaria is characterized by an absence of clinical symptoms of the disease despite the presence of *Plasmodium* sp. in the bloodstream. The global strategy for malaria control relies mainly on early detection and appropriate treatment [1, 2]. People with asymptomatic *Plasmodium* sp. infection are often not diagnosed and treated, although these infections can evolve into symptomatic malaria or serve as a source of infection for malaria vector mosquitoes, thus contributing to the continued transmission of the disease [3, 4]. In sub-Saharan Africa, studies have demonstrated that asymptomatic infections are more prevalent among children and pregnant women, who are particularly vulnerable populations [5, 6]. While great progress has been made in malaria control, elimination has proven challenging due to the persistence of asymptomatic infections that act as parasite reservoirs. Understanding the burden of asymptomatic malaria is critical for targeting interventions to interrupt transmission and achieve elimination. This type of study can provide key insights into the prevalence of asymptomatic infections in different epidemiological settings. By using highly sensitive molecular techniques, the detection of submicroscopic parasite densities that are undetectable by conventional diagnostics is possible. This allows quantification of the asymptomatic reservoir and assessment of its contribution to ongoing transmission [7]. Such data enables the tailoring of mass drug administration and other interventions to clear both symptomatic and asymptomatic infections in a population. Overall, this data can provide evidence to guide strategies aimed at tackling the asymptomatic parasite reservoir, which is vital for progressing towards malaria elimination globally [8].

In Gabon, malaria transmission is perennial due to its hot and humid equatorial climate favorable to mosquito proliferation. Rural areas are more affected than urban regions, primarily due to environmental conditions that promote mosquito proliferation, and *P. falciparum* is responsible for 96%–100% of infections [9, 10]. With an estimated population of 2,484,789, malaria remains the primary cause of consultations and hospital admissions in Gabon, with 535,939 cases reported in 2021, corresponding to an incidence rate of 228.9 cases per 1000 residents. In the same year, the disease was responsible for an estimated 384 deaths [11]. In the country, malaria cases rise significantly during the rainy season (October to mid-December and March to June). As in all sub-Saharan African countries, malaria is a public health problem in the country despite the control measures implemented by the Ministry of Health of Gabon through the National Malaria Control Program (NMCP). Gabon's NMCP aims to reduce the incidence and mortality associated with this disease by implementing an integrated strategy focusing on (i) prevention through the distribution of insecticide-treated nets and indoor residual spraying, (ii) early diagnosis and effective treatment of cases through training and improved access to care, (iii) strengthening surveillance systems to monitor trends, (iv) raising community awareness, and (v) partnerships to maximize impact. In Gabon, asymptomatic *Plasmodium* infections have been less studied compared to symptomatic infections. A few studies on asymptomatic malaria have been conducted among school-age children and pregnant women [12–14], and no data exist regarding the general population (all ages combined). Therefore, this study was aimed at determining the microscopic prevalence of *Plasmodium* sp. infection and its associated factors among people living in Franceville (urban area) and in the villages of Pana and Mvengue.

## 2. Methods

### 2.1. Study Area and Period

**Figure 1:**
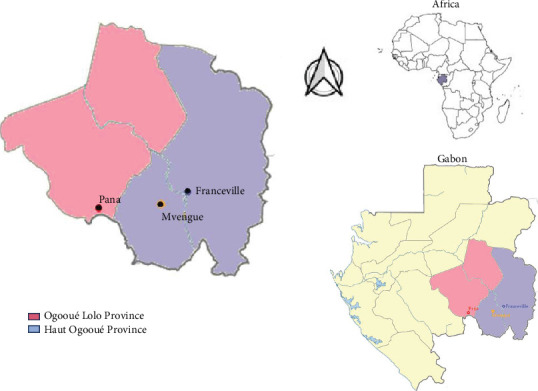
Selection sites.

This was a cross-sectional study conducted among people residing in urban areas (Franceville) and rural areas (Mvengue and Pana) (Figure 1) between April and July 2022. Franceville is an urban city located in the Haut-Ogooué Province of Gabon. It is the third-largest town in Gabon in terms of population. The population of Franceville is approximately 110,568 inhabitants [15]. In Franceville, the primary malaria vectors in this region are *Anopheles funestus* and *Anopheles gambiae*. Malaria remains the leading cause of medical visits and hospitalizations, despite some fluctuations in incidence over recent years. A study from 2017 to 2019 showed a decrease in clinical cases, yet the disease burden remains high. Severe malaria cases, particularly those caused by *P. falciparum*, peak during the rainy season (Imboumy et al. 2023). The village cluster of Mvengue is a rural community in the Haut-Ogooué Province with a population of approximately 2000 inhabitants, according to the most recent population census conducted in 2020 [15]. Pana is the prefecture of the Lombo-Bouenguidi department, located in the Ogooué Lolo Province. This village has poor economic development; it is surrounded by forest, and it is located at an altitude of around 400 m above sea level. This village has approximately 1579 inhabitants [15].

### 2.2. Study Design and Population

This community-based, cross-sectional, descriptive study was conducted from April to July 2022. The study population was composed of willing inhabitants of all ages. Inclusion criteria stipulated that participants must reside in the study site during the study. It was a *Plasmodium* sp. detection campaign. A minimum of 323 participants for each area (urban and rural) was calculated using the following formula to have the appropriate sample size: *n*=𝑍^2^⁣^∗^(1‐𝑃)/𝑑2. *n* is the sample size, *Z* is the statistic corresponding to the confidence level (*Z* = 1.96), and *P* is the estimated prevalence of asymptomatic malaria in Gabon (30%).

Prior to the screening campaigns, the staff involved in this study (clinicians, laboratory technicians, researchers, nurses, and community health workers) were trained on the study objectives and procedures, including obtaining informed consent, administering the questionnaire, measuring axillary temperature and weight, and preparing thick and thin blood smears, before data collection. Mobilization of the participating communities was organized through radio announcements and banners for the urban populations (Franceville) and through information campaigns for the rural populations (Pana and Mvengue) before the start of the study. Then, mobile health clinics were set up at strategic points in the city of Franceville and, for rural areas, in dispensaries and village chiefs' locations (Pana and Mvengue).

### 2.3. Procedures for Data Collection

The survey questionnaire was designed in French, the national language in Gabon, and translated into local languages (Nzebi, Obamba, and Téké languages) by the interviewers when necessary. The surveys started with a short presentation of the project, and we asked participants to say whether they agreed to participate in the study voluntarily. Then sociodemographic data and their attitude and practice on malaria were collected. We conducted in-person interviews, approximately 30 min in length.

To determine their attitude and practice on malaria, some key questions were asked, such as: “Do you use a malaria prevention method?”, if yes, which one: long-lasting insecticide-treated nets (LLINs), repellent? Insecticide? “Do you practice self-medication?”, if yes, which medication do you use? The questionnaire was completed and signed by the investigator and the participant or parent/guardian of the participant for minors, after being informed.

### 2.4. Malaria Screening and Treatment

Malaria screening was performed on volunteers of all ages after obtaining informed consent. *Plasmodium* sp. infection was performed by microscopy according to the Lambarene method [16]. Briefly, 10 *μ*L of blood is evenly distributed on a 10 × 18 mm area of a microscope slide (drawn on paper underneath the slide) with a micropipette. The slide is dried and stained with 20% Giemsa for 10 min and then examined under the microscope. Parasitaemia is calculated by dividing the number of parasites counted by the number of fields read (usually 10 fields) and multiplying the result by an appropriate multiplication factor that depends on the magnification and the area of the microscopic field. For our microscope, the multiplication factor is 750. All blood smears were examined by two experienced technicians. In the event of divergent results, a third qualified reader re-examined the slide and carried out an internal quality control on 10% of the slides. The final result is the arithmetic mean of the findings from the two technicians. Both clinical and anthropometric parameters were recorded on a patient sheet, and data on symptoms were also collected. Blood smears were read by experienced microscopists, and results were provided within 45 min to 1 h after sampling.

Participants with positive results were offered treatment with either artemether–lumefantrine (Coartem, Novartis, Switzerland) for children and pregnant women in their second and third trimesters, or quinine for pregnant women in their first trimester, Surquina 250 mg (Innothera Chouzy, France), according to national treatment guidelines.

### 2.5. Operational Definitions

i. Asymptomatic malaria: absence of malaria-related symptoms in the past 2 days and at the time of the survey, with the presence of malaria parasites in the blood [17].
ii. Income-generating activities (IGAs): individuals engaged in public or private sector work, or any initiatives (commerce and small business) producing regular income.
iii. Repellents: any means to limit or prevent mosquitoes from entering the house.

### 2.6. Ethical Considerations

This study was authorized by the Ministry of Health, the National Research Ethics Committee of Gabon (No. 0013/2022/CNER/P/SG), and the South-East Regional Health Directorate of the Gabonese Republic (1784/MS/SG/DRSSE). Participation was voluntary after informed consent, and the reporting of results was strictly confidential.

### 2.7. Data Analysis

All data were recorded in Excel 2013 spreadsheets. Statistical analysis was performed using R Version 4.0.5 (2021-03-31) software. Qualitative variables were described as proportions and quantitative variables as means, standard deviations (SDs), and medians with interquartile ranges (IQRs). Proportions of qualitative variables were compared using a nonparametric chi-squared test or Fisher's exact test for numbers less than 5. Crude odds ratios (ORs) are presented, and adjusted odds ratios (aORs) of independent risk factors were calculated using binary logistic regression models. The confidence interval was set at 95% (95% confidence interval (CI)). Statistical significance was set at *α* = 5%.

## 3. Results

### 3.1. Sampling Description

A total of 976 participants were included in this study, including 404 men and 572 women for a sex ratio (M/F) of 0.71. Among these participants, 491 were recruited in urban areas (Franceville) and 485 in rural areas (163 in Pana and 322 in Mvengue). Among the female participants, 4.90% (28/572) were pregnant. The sociodemographic, anthropometric parameters, and clinical characteristics of the participants are shown in Table 1. Almost all 94.74% (925/976) participants were asymptomatic, and the remaining participants (17 in urban areas and 34 in rural areas) had fever or a history of fever within 48 h prior to screening. The age, weight, and average temperature of the participants were higher among those residing in urban areas (*p* < 0.001). In urban areas, participants over 23 years of age were the most numerous, while in rural areas, those aged 0–5 years were the majority (*p* < 0.001). Participants with IGAs were more numerous in urban than in rural areas (*p* < 0.001).

### 3.2. Attitude and Practice Towards Malaria

Of the 976 people screened, 784 took part in the survey on attitudes and practices regarding malaria, representing 80.33% of the participants. Among them, 491 were located in urban areas, while 293 were in rural areas. The average age was 33.6 ± 20.2 years for urban participants and 30.2 ± 17.8 years for those residing in rural areas (*p* = 0.014). This study shows that attitudes and practices towards malaria differ between urban and rural regions (Table 2). Most participants residing in rural areas (64%) sleep under a LLIN, in contrast to those (34%) living in urban areas (*p* < 0.001). The use of repellents and insecticides was more widespread among participants living in rural areas. In urban areas, participants reported more recourse to self-medication in case of fever (Table 2), and antimalarials are the most widely used drugs.

### 3.3. General Characteristics of Malaria Infection

The overall prevalence of *Plasmodium* infection was 21.62% (211/976; 95%CI [19.15–24.31]). The mean parasitemia and temperature were 1945 ± 6725 parasites per microliter and 36.1°C ± 0.90°C, respectively. The average weight of infected participants (39.4 ± 27.5 kg) was significantly lower (*p* < 0.001) than that of uninfected participants (49 ± 30.4). In total, 4.74% (10/211) of screened participants had a fever or a history of fever: three in urban areas and seven in rural areas. In urban areas, the prevalence of *Plasmodium* infection was 19.35% (95/491; 95%CI [16.10–23.07]). The average parasitemia was 2726 ± 5937 parasites per microliter. *Plasmodium malariae* and *P. falciparum* were responsible for 2.11% and 96.84% of infections, respectively. The coinfection of *P. falciparum* and *P. malariae* represented 1.05% of infections. In rural areas, the prevalence of *Plasmodium* infection was 23.92% (116/485, 95%CI [20.34–27.91]); *P*. *falciparum* was responsible for 100% of infections, and 93.97% (109/116) of them were asymptomatic. The prevalence of infection in rural areas (23.92%) was statistically the same (*p* = 0.083) as that in urban areas (19.34%).

### 3.4. Association of Socioeconomic, Attitude, and Practice Factors With Asymptomatic Malaria Infection in Urban Area

In urban areas, fever was not associated with *Plasmodium* infection (OR = 1.12; 95%CI = [0.32‐3.99]). The average weight (55.4 ± 26.6 kg) and average temperature (37.4°C ± 1.09°C) of infected participants were not different from those of uninfected participants (60.1 ± 28 and 37.4 ± 1.05 for the weight and temperature of uninfected participants, respectively). The most infected age group (aOR = 3.75; 95%CI = [1.15‐12.1]; *p* = 0.031) was 18–23 years (Table 3). This study does not show a link between sociodemographic characteristics, attitudes, and practices with asymptomatic *Plasmodium* infection (Table 3).

### 3.5. Association of Socioeconomic, Attitude, and Practice Factors With Asymptomatic Malaria Infection in Rural Area

In rural areas, fever was not associated with *Plasmodium* infection (OR = 1.22, 95%CI = [0.52‐2.90]). The prevalence of asymptomatic *Plasmodium* infection differed according to age groups (*p* < 0.001). It was higher in children aged 6–11 years (Table 4). The risk of infection was lower in adults over 23 years of age (aOR = 0.28; 95%CI = [0.14‐0.51]; *p* < 0.001). The prevalence of infection (32.95%) was highest in children aged 6–11 years (Table 4). Socioeconomic characteristics, attitudes, and practices towards *Plasmodium* infection were not associated with a risk of asymptomatic malaria infection (Table 4).

## 4. Discussion

Malaria remains a major public health concern in developing countries despite a significant decrease in prevalence in almost all endemic areas. The global strategy for controlling this disease relies mainly on early detection and appropriate treatment of *Plasmodium* infection [1]. In this work, we determined for the first time the microscopic prevalence of *Plasmodium* infection in the general population and its associated factors among people living in urban (Franceville) and rural (Pana and Mvengue) areas in south-east Gabon.

The results of this study highlight a high prevalence of microscopic *Plasmodium* infection in the general population, both in urban (19.35%) and rural (23.92%) areas, with no significant difference (*p* = 0.083) between the two settings. These prevalences are comparable to those reported in other sub-Saharan African countries, where the prevalence of *Plasmodium* infection in the population can vary from 12% to 75% depending on the regions and populations studied [18, 19]. However, the prevalences observed in our study are higher than that recently reported in Franceville (rural area) and lower than that found in Lastoursville (rural area) by Kouna et al. among school-age children screened in primary schools [14]. This difference could be explained by the age groups included in the two studies, as our study included participants of all ages. Our data confirm that *P. falciparum* is responsible for almost all malaria infections in Gabon (96.84% in urban areas and 100% in rural areas), consistent with the country's epidemiological data [13, 20]. Interestingly, the majority of detected infections were asymptomatic, both in urban (96.84%) and rural (93.97%) areas. This result underscores the importance of the asymptomatic carrier reservoir in malaria transmission [7, 8]. Indeed, asymptomatic malaria plays an important role in the continuous transmission of the disease. This epidemiological study seems to show that a significant number of malaria infections in the general population of south-east Gabon are asymptomatic, highlighting the importance of active surveillance and screening of asymptomatic carriers in malaria control and elimination strategies in the country. Their detection and treatment could be an important lever for disease control and elimination [12].

This study showed differences in the age distribution of infection between urban and rural areas. In urban areas, the most affected age group was 18–23 years, while in rural areas, it was children aged 6–11 years. These results are consistent with those of other studies conducted in Africa, which have shown that the prevalence of asymptomatic infection tends to be higher among school-age children in rural areas, due to more frequent exposure to mosquito bites and a lower level of acquired immunity [21, 22]. In contrast, in urban areas, young adults may be more exposed due to their professional or leisure activities in the evening, when mosquitoes are most active [22, 23]. Also, no significant association was found between sociodemographic characteristics, attitudes, and practices towards malaria and the risk of asymptomatic infection, both in urban and rural areas. This finding contrasts with the results of other studies that have highlighted the importance of factors such as IGAs and the use of repellents or insecticide-treated nets in malaria prevention and control [24, 25]. It is possible that our study did not have sufficient statistical power to detect these associations due to the sample size and the low prevalence of certain risk factors in the studied population.

In this study, although fever was not associated with *Plasmodium* infection in either urban or rural settings (Tables 3 and 4), several participants reported resorting to self-medication with antimalarials in case of fever, especially in urban areas (Table 2). This could be explained by the fact that the population often associates fever with malaria. Furthermore, these results demonstrate once again that self-medication remains a common practice, particularly in urban areas where access to over-the-counter medications is easier [26, 27]. This underscores the importance of strengthening public education on the risks of self-medication and promoting medical consultation in cases of suspected malarial fever.

Among the limitations of this study is the absence of detection of submicroscopic infections by molecular techniques (such as polymerase chain reaction), which could have revealed an even higher prevalence of asymptomatic carriage [7]. Moreover, the cross-sectional design does not allow for the evaluation of the temporal dynamics of infections and their potential long-term clinical impact. Finally, environmental factors (e.g., type of housing and proximity to larval breeding sites) were not taken into account and could modulate the risk of infection.

## 5. Conclusion

This study highlights a high prevalence of microscopic *Plasmodium* infection in the general population of south-east Gabon, with differences between urban and rural areas in terms of age distribution. These results underscore the need to adapt malaria control strategies to the different epidemiological contexts of the country, taking into account the specificities of at-risk populations and local determinants of transmission.

## Figures and Tables

**Table 1:** General characteristics of participants in urban and rural areas.

**General characteristics**	**All participants**	**Regions**		**p** ** value**
		**Urban**	**Rural**	
*N* (number)	976	491	485	—
Age (in years)				
Mean (SD)	25.70 (21.4)	33.6 (20.2)	17.6 (19.5)	**< 0.001**
Median [IQR]	22 [6–42]	35 [15–49]	10.0 [4–25]	—
Weight (in kg)				
Mean (SD)	46.7 (30)	59.2 (27.8)	25.1 (19.7)	**< 0.001**
Median [IQR]	49 [16–70]	62 [42–80]	17 [12–32.8]	—
Temperature (in °C)				
Mean (SD)	37.1 (0.94)	37.4 (1.05)	36.7 (0.51)	**< 0.001**
Median [IQR]	37 [36.6–37.5]	37.4 [36.7–37.4]	36.7 [36.5–36.9]	—
Sex				
Female, *n* (%)	572 (58.61)	281 (57)	291 (60)	0.38
Male, *n* (%)	404 (41.39)	210 (43)	194 (40)	
Age groups (in years)				
0–5, *n* (%)	240 (24.59)	71 (14)	169 (35)	**< 0.001**
6–11, *n* (%)	126 (12.91)	38 (7.7)	88 (18)	
12–17, *n* (%)	86 (8.81)	20 (4.1)	66 (14)	
18–23, *n* (%)	54 (5.53)	17 (3.5)	37 (7.6)	
> 23, *n* (%)	470 (48.16)	345 (70)	125 (26)	
IGAs				
No	606 (78.29)	330 (67)	276 (94)	**< 0.001**
Yes	168 (21.71)	161 (33)	17 (5.8)	

*Note:* Values in bold indicate statistically significant differences.

Abbreviation: IGAs: income-generating activities.

**Table 2:** Attitudes and practices towards malaria infection in urban and rural regions.

	**Region, ** **n** ** (%)**		**p** ** value**
	**Urban**	**Rural**	
LLINs			
No	322 (66)	106 (36)	**< 0.001**
Yes	169 (34)	187 (64)	—
Repellent			
None	446 (91)	124 (42)	**< 0.001**
Mosquito coil	36 (7.3)	169 (58)	—
Household fan	8 (1.6)	0 (0)	—
AC	1 (0.2)	0 (0)	—
Insecticide			
No	419 (85)	124 (42)	**< 0.001**
Yes	72 (15)	169 (58)	—
Self-medication			
No	348 (70.29)	248 (83.78)	**< 0.001**
Yes	140 (28.69)	48 (16.22)	—
Medication administered			
Antimalarial	121 (86.43)	33 (68.75)	**0.003**
Antipyretic	14 (10)	15 (45.45)	—
Anti-inflammatory	3 (2.14)	0 (0)	—
Antibiotic	2 (1.43)	0 (0)	—

*Note:* Values in bold indicate statistically significant differences.

Abbreviation: AC: air conditioning.

**Table 3:** Association of socioeconomic, attitude, and practice factors with asymptomatic malaria infection in urban regions.

	**n**	**Prevalence of infection, ** **n** ** (%)**	**Univariate**	**Multivariate**	**p** ** value**
			**OR (95% CI)**	**aOR (95% CI)**	
Sex					
Female	281	59 (21)	1		
Male	210	36 (17.14)	0.77 (0.49–1.23)		
Age groups					
0–5	71	13 (18.31)	1	1	Ref
6–11	38	12 (31.58)	1.74 (0.3–3.65)	2.44 (0.95–6.36)	0.063
12–17	20	5 (25)	1.25 (0.44–3.5)	1.81 (0.51–5.87)	0.33
18–23	17	7 (41.18)	2.63 (0.96–7.21)	3.75 (1.15–12.1)	**0.026**
> 23	345	58 (16.81)	0.76 (0.51–1.14)	1.13 (0.55–2.48)	0.75
FhF					
Yes	17	3 (17.65)	1		
No	474	92 (19.41)	1.12 (0.32–3.99)		
IGAs					
No	330	70 (21.21)	1	1	Ref
Yes	161	25 (15.53)	0.68 (0.41–1.13)	0.75 (0.41–1.32)	0.32
LLINs					
No	323	65 (20.12)	1	1	Ref
Yes	170	26 (15.29)	0.72 (0.44–1.18)	0.69 (0.41–1.13)	0.15
Repellent					
None	447	86 (19.24)	1		
Mosquito coil	37	5 (13.51)	0.66 (0.25–1.73)		
Household fan	8				
AC	1				
Insecticide					
No	421	76 (18.05)	1		
Yes	72	15 (20.83)	1.19 (0.64–2.22)		
Self-medication					
No	353	58 (16.43)	1		
Yes	140	33 (23.57)	1.57 (0.97–2.54)		

*Note:* Values in bold indicate statistically significant differences.

Abbreviations: AC: air conditioning; FhF: fever or history of fever; IGAs: income-generating activities.

**Table 4:** Association of sociodemographic, attitude, and practice factors with malaria infection in rural regions.

	**n**	**Asymptomatic infection, ** **n** ** (%)**	**Univariate**	**Multivariate**	**p** ** value**
			**OR (95% CI)**	**aOR (95% CI)**	
Sex					
Female	291	68 (23.37)	1		
Male	194	48 (24.74)	1.08 (0.71–1.65)		
Age groups					
0–5	169	53 (31.36)	1	1	Ref
6–11	88	29 (32.95)	1.08 (0.62–1.87)	1.08 (0.62–1.86)	0.8
12–17	66	11 (16.67)	0.44 (0.21–0.90)	0.44 (0.20–0.89)	**0.026**
18–23	37	9 (24.32)	0.70 (0.31–1.59)	0.70 (0.29–1.56)	0.4
> 23	125	14 (11.29)	0.28 (0.15–0.53)	0.28 (0.14–0.51)	**< 0.001**
FhF					
Yes	34	7 (20.59)	1		
No	451	109 (24.17)	1.22 (0.52–2.90)		
IGAs					
No	274	43 (15.69)	1		
Yes	17	3 (17.65)	1.15 (0.32–4.18)		
LLINs					
No	105	16 (15.24)	1		
Yes	186	30 (16.13)	1.07 (0.55–2.07)		
Repellent					
None	168	15 (8.92)	1	1	Ref
Mosquito coil	123	31 (25.20)	3.43 (1.76–6.70)	0.66 (0.33–1.29)	0.23
Insecticide					
No	122	17 (13.93)	1		
Yes	169	29 (17.16)	1.28 (0.68–2.45)		
Self-medication					
No	243	38 (15.64)	1		
Yes	48	8 (16.67)	1.07 (0.47–2.49)		

*Note:* Values in bold indicate statistically significant differences.

Abbreviations: FhF: fever or history of fever; IGAs: income-generating activities.

## Data Availability

The data that support the findings of this study are available on request from the corresponding author. The data are not publicly available due to privacy or ethical restrictions.

## References

[1] WHO (2023). *World malaria report 2022*.

[2] Whittaker C., Slater H., Nash R. (2021). Global patterns of submicroscopic *Plasmodium falciparum* malaria infection: insights from a systematic review and meta-analysis of population surveys. *The Lancet Microbe*.

[3] Biruksew A., Demeke A., Birhanu Z., Golassa L., Getnet M., Yewhalaw D. (2023). Schoolchildren with asymptomatic malaria are potential hotspot for malaria reservoir in Ethiopia: implications for malaria control and elimination efforts. *Malaria Journal*.

[4] Alves F. P., Gil L. H., Marrelli M. T., Ribolla P. E., Camargo E. P., Da Silva L. H. (2005). Asymptomatic carriers of *Plasmodium spp*. as infection source for malaria vector mosquitoes in the Brazilian Amazon. *Journal of Medical Entomology*.

[5] Korzeniewski K., Bylicka-Szczepanowska E., Lass A. (2021). Prevalence of asymptomatic malaria infections in seemingly healthy children, the rural Dzanga Sangha region, Central African Republic. *International Journal of Environmental Research and Public Health*.

[6] Kassie G. A., Azeze G. A., Gebrekidan A. Y. (2024). Asymptomatic malaria infection and its associated factors among pregnant women in Ethiopia; a systematic review and meta-analysis. *Parasite Epidemiology and Control*.

[7] Essone J.-C. B. B., Imboumy-Limoukou R.-K. (2022). Infection submicroscopique à Plasmodium falciparum en zone d’endémie palustre : une revue de littérature. *Annals of African Medicine*.

[8] Ibrahim A. O., Bello I. S., Ajetunmobi A. O., Ayodapo A., Afolabi B. A., Adeniyi M. A. (2023). Prevalence of asymptomatic malaria infection by microscopy and its determinants among residents of Ido-Ekiti, Southwestern Nigeria. *PLoS ONE*.

[9] Maghendji-Nzondo S., Nzoughe H., Lemamy G. J. (2016). Prevalence of malaria, prevention measures, and main clinical features in febrile children admitted to the Franceville Regional Hospital, Gabon. *Parasite*.

[10] Maghendji-Nzondo S., Kouna L. C., Mourembou G. (2016). Malaria in urban, semi-urban and rural areas of southern of Gabon: comparison of the Pfmdr 1 and Pfcrt genotypes from symptomatic children. *Malaria Journal*.

[11] WHO (2023). *Country disease outlook Gabon*.

[12] Nkoghe D., Akue J. P., Gonzalez J. P., Leroy E. M. (2011). Prevalence of *Plasmodium falciparum* infection in asymptomatic rural Gabonese populations. *Malaria Journal*.

[13] Biteghe-Bi-Essone J. C., Imboumy-Limoukou R. K., Ekogha-Ovono J. J. (2022). Intermittent preventive treatment and malaria amongst pregnant women who give birth at the Centre Hospitalier Régional Paul Moukambi de Koula-Moutou in southeastern Gabon. *Malaria Journal*.

[14] Kouna L. C., Oyegue-Liabagui S. L., Voumbo-Matoumona D. F., Lekana-Douki J. B. (2024). Malaria prevalence in asymptomatic and symptomatic children living in rural, semi-urban and urban areas in eastern Gabon. *Acta Parasitologica*.

[15] LIBREVILLE DGDLSD (2015). Recensement Général de la Population et des Logements de 2013 du Gabon. https://instatgabon.org/uploads/folder_1/RESULTATS-GLOBAUX-RGPL2013-OK_DECEMBRE-2015-1.pdf.

[16] Planche T., Krishna S., Kombila M. (2001). Comparison of methods for the rapid laboratory assessment of children with malaria. *The American Journal of Tropical Medicine and Hygiene*.

[17] Zerdo Z., Bastiaens H., Anthierens S. (2021). Prevalence and associated risk factors of asymptomatic malaria and anaemia among school-aged children in Dara Mallo and Uba Debretsehay districts: results from baseline cluster randomized trial. *Malaria Journal*.

[18] Berzosa P., de Lucio A., Romay-Barja M. (2018). Comparison of three diagnostic methods (microscopy, RDT, and PCR) for the detection of malaria parasites in representative samples from Equatorial Guinea. *Malaria Journal*.

[19] Okell L. C., Ghani A. C., Lyons E., Drakeley C. J. (2009). Submicroscopic infection in *Plasmodium falciparum*-endemic populations: a systematic review and meta-analysis. *The Journal of Infectious Diseases*.

[20] Lendongo-Wombo J. B., Oyegue-Liabagui S. L., Biteghe-Bi-Essone J. C., Ngoungou E. B., Lekana-Douki J. B. (2022). Epidémiology of malaria from 2019 to 2021 in the southeastern city of Franceville, Gabon. *BMC Public Health*.

[21] Walldorf J. A., Cohee L. M., Coalson J. E. (2015). School-age children are a reservoir of malaria infection in Malawi. *PLoS One*.

[22] Nankabirwa J., Brooker S. J., Clarke S. E. (2014). Malaria in school-age children in Africa: an increasingly important challenge. *Tropical Medicine & International Health*.

[23] Ngasala B., Mwaiswelo R. O., Chacky F. (2023). Malaria knowledge, attitude, and practice among communities involved in a seasonal malaria chemoprevention study in Nanyumbu and Masasi districts, Tanzania. *Frontiers in Public Health*.

[24] Tusting L. S., Willey B., Lucas H. (2013). Socioeconomic development as an intervention against malaria: a systematic review and meta-analysis. *Lancet*.

[25] Degarege A., Fennie K., Degarege D., Chennupati S., Madhivanan P. (2019). Improving socioeconomic status may reduce the burden of malaria in sub Saharan Africa: a systematic review and meta-analysis. *PLoS One*.

[26] Iribhogbe O. I., Odoya E. M. (2021). Self-medication practice with antimalarials & the determinants of malaria treatment-seeking behavior among postpartum mothers in a rural community in Nigeria. *Pharmacoepidemiology and Drug Safety*.

[27] Akilimali A., Bisimwa C., Aborode A. T. (2022). Self-medication and anti-malarial drug resistance in the Democratic Republic of the Congo (DRC): a silent threat. *Tropical Medicine and Health*.

